# Generation and characterization of MEK and ERK inhibitors- resistant non-small-cells-lung-cancer (NSCLC) cells

**DOI:** 10.1186/s12885-018-4949-6

**Published:** 2018-10-23

**Authors:** Alice Iezzi, Elisa Caiola, Arianna Scagliotti, Massimo Broggini

**Affiliations:** Laboratory of Moleular Pharmacology, Istituto di Ricerche Farmacologiche Mario Negri IRCCS, Milan, Italy

**Keywords:** NSCLC, Drug resistance, MEK inhibitors, ERK inhibitors

## Abstract

**Background:**

The RAS/RAF/MEK/ERK pathway is one of the most downregulated pathway in cancer. Inhibitors of RAF and MEK have established clinical use while ERK inhibitors recently faced the clinic. We aimed to generate resistant cell lines which could be helpful for defining new combinations able to overcome resistance.

**Methods:**

the human NSCLC cell line NCI-H727, sensitive to both MEK and ERK inhibitors, was treated with increasing concentrations of MEK162 (as MEK inhibitor) or SCH772984 as ERK inhibitor.

**Results:**

we successfully obtained a MEK resistant subline (H727/MEK, after 40 passages) as well as an ERK resistant subline (H727/SCH, after 18 passages). The two resistant sublines H727/MEK and H727/SCH were cross-resistant to ERK and MEK inhibitors, respectively, but not to RAF inhibitors. The sublines maintained the responsiveness to inhibitors of the parallel PI3K/akt/mTOR pathway as well as to agents with different mechanism of action. Mechanistically, treatment of sensitive and resistant cells with MEK or ERK inhibitors was able to induce a similar inhibition of ERK phosphorylation, while only in parental cells the drugs were able to induce a downregulation of S6 and RSK phosphorylation.

**Conclusions:**

these resistant cells represent an important tool for further studies on the mechanisms of resistance and ways to overcome it.

## Background

The RAS/RAF/MEK/ERK pathway is one of the most downregulated pathway in cancer [1–4].

Among the proteins belonging to this pathway, KRAS is the only one for which no drugs directly targeting its activity are available. B-RAF, MEK and ERK inhibitors are available and some already marketed for specific indications. Vemurafenib (B-RAF inhibitor) has been approved for the treatment of late stage and BRAF mutated melanoma as well as for the treatment of Erdheim-Chester Disease (a rare histiocytosis marked by recurrent BRAFV600E mutations in more than half of patients). Another B-RAF inhibitor, dabrafenib received approval for the treatment of BRAF mutated melanoma while trametinib, a MEK inhibitor has been approved for the same indication, as well as for the treatment of B-Raf mutated NSCLC, both as single agent and in combination with dabrafenib. For ERK, an inhibitor, Ulixertinib, has recently entered clinical investigation as first in class drug [5].

Both MEK and, particularly, B-RAF inhibitors have shown activity in NSCLC tumors with altered expression of mutational status of their target, although in several instances the good response was limited by the appearance of acquired resistance [2, 6–9].

Several mechanisms associated to resistance development have been reported [10–17]. They go to changes in target splicing, upregulation, changes in phosphorylation of downstream proteins, different metabolism modulation.

To try to better elucidate the mechanisms associated to resistance to RAS/RAF/MEK/ERK pathway inhibitor and to try to overcome the resistance, it is mandatory to have preclinical models of resistance.

Being MEK/ERK inhibitors a reasonable option for the treatment of NSCLC [7], we developed, starting from a human NSCLC cell lines sensitive to MEK and ERK inhibitors, two distinct sublines resistant to MEK-162 (MEK inhibitor) and SCH772984 (ERK inhibitor), respectively. Here we report the initial molecular and pharmacological characterization of these cells, which will be helpful for the development of alternative treatments or combinations.

## Methods

### Cells

The human non small cell lung cancer cell lines NCI-H727 (CRL-5815), responsive to both MEK and ERK inhibitors treatments in vitro, NCI-H460 (HTB-177), A549 (CCL-185) and NCI-H1299 (CRL-5083), originally obtained from ATCC, were maintained in RPMI1640 medium supplemented with 10% FBS. The cells were routinely checked for the absence of mycoplasma and authenticated using short tandem repeat (STR) method. The STR profiles were compared with the American Type Culture Collection database or the German Collection of Microorganisms and Cell Cultures database.

The NCI-H727cell line has been treated with increasing concentrations of the MEK inhibitor MEK162 or the ERK inhibitor SCH772984 for several passages in vitro until development of resistance.

The MTS assay (Promega) was used to determine the activity of drugs in vitro as described [18]. The drugs used in the study were: ulixertinib, GDC0994, cisplatin, olaparib, docetaxel, BYL-719, torin-1, doxorubicin. All these drugs were obtained from Selleckchem. Stock solutions were prepared in DMSO for all the drugs except doxorubicin and cisplatin which were dissolved in water and culture medium, respectively. Dilutions from stock solutions were performed in culture medium. Concentrations dependent curves were plotted as percentages relative to untreated controls, with at least six replicates for each time point. The mean and SD of at least three independent experiments are presented. Concentrations inhibiting the growth by 50% (IC50) were calculated from the curves using Graphpad Prism Version 7.

### Western blotting analyses

Proteins were extracted from exponentially growing cells and visualized as described [18]. Immunoblotting was carried out with the following antibodies: anti-S6 (Ser235/236) ribosomal protein #2211, anti-S6 ribosomal protein #2217, anti-p90RSK #9355, anti-p90RSK (Thr 359/Ser363) #9344 provided by Cell Signalling Technology. Anti-ERK #sc94, anti-ERK (Tyr204) #sc7383, were obtained from Santa Cruz Biotechnology.

### Real-time RT PCR

RNA was extracted by using Maxwell 16LEV simplyRNA Cells kit (Promega). RNA was retro-transcribed to cDNA using High Capacity cDNA Reverse Transcription Kit (Life Technologies). MDR-1 gene expression was determined by real-time RT-PCR performed with Sybr Green PCR master mix (Promega), evaluating for each gene the dissociation curve. Values were normalized using the expression of the housekeeping gene actin and the levels in resistant cells compared to those of parental cells. Real-time PCR was done using the 7900HT Sequence Detection System (Applied Biosystems).

## Results

### Development of MEK 162 and SCH772984 resistance

After ten passages in vitro in the presence of 50 (for the first 3 passages) or 100 nM (for the remaining 7 passages) of the drug, we could detect a reduced response to MEK162 treatment (resistance index 10). The maintenance of these cells in drug-free medium, however, completely reverted, after six passages, the resistance. The cells were therefore re-challenged with MEK162 and the treatments were continued for additional 30 passages. The cells, after a total of 40 passages in the presence of drug (which was increased up to 600 nM) showed a resistance index of approximately 10 (the IC50 in the parental cell line was approximately 115 nM while in the resistant clone the IC50 was estimated > 1000 nM) (Table 1). This resistance was maintained after several passages in vitro in drug free medium and we considered these cells (H727/MEK) stably resistant to the drug.

**Table 1:** IC50 values of SCH772984 and MEK162 in parental and resistant cells

	IC50 MEK162 (nM)	RI	IC50 SCH772984 (nM)	RI
H727	115	–	135	–
H727-MEK	> 1000	> 9	> 1000	> 9
H727-SCH	> 1000	> 9	> 1000	> 9

For the ERK inhibitor SCH772984, a resistance index of approximately 10 was obtained already after nine passages in the presence of the inhibitor. This resistance was maintained in further drug-free medium passages. The pressure of drug was anyway maintained for additional nine passages and at passage 18 the treatments were stopped. At this stage the IC50 of SCH772984 in these cells (H727/SCH) was not reached and was estimated as > 1000 nM, while the drug showed an IC50 of 135 nM in the parental cells (Table 1).

The table also shows the activity of MEK162 against H727/SCH cells and of SCH772984 against H727/MEK cells. As it can be seen, MEK resistant cells are also resistant to ERK inhibitor and SCH resistant cells are resistant to MEK inhibitor.

The two H727-resistant cell lines were then tested for cross-resistance with other ERK inhibitors.

**Fig. 1:**
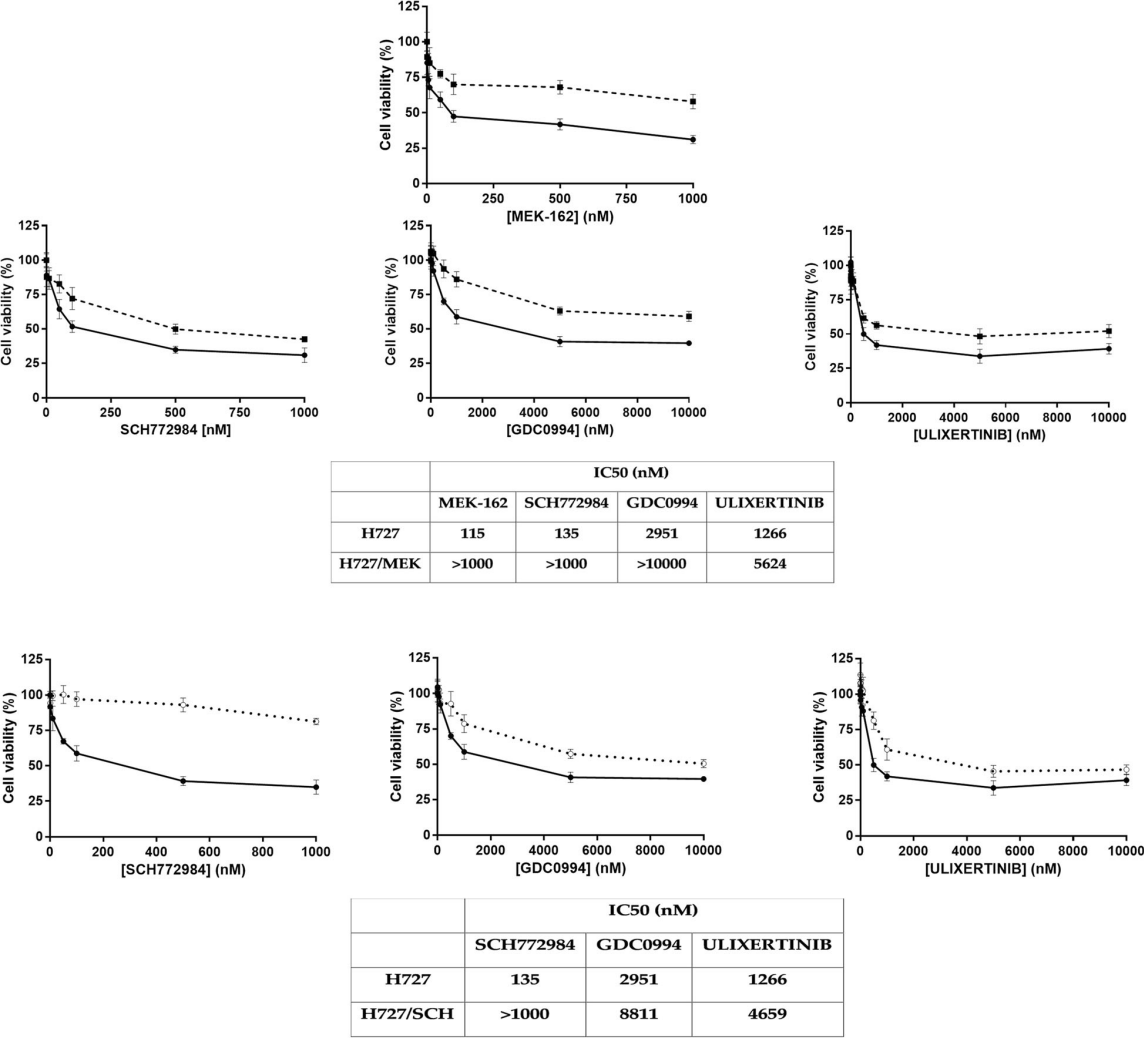
Activity of SCH772984 and two additional ERK inhibitors (GDC0994 and ulixertinib) in H727/MEK (Upper panel) and H727/SCH (Lower panel) cells. Each graph reports the concentration dependent inhibition curves in resistant (○∙∙∙∙○) and parental (●—●) cells. For each graph the concentration of the specifi inhibitor are reported in the X axis, while the percentage of inhibition is reported in the Y axis. The values below the graphs are the calculated IC50 for each compound in each cell line

As shown in Fig. 1 panel a, H727/SCH cells were also (although at lower level) resistant to other ERK inhibitors (GDC0994 and ulixertemib). Similarly, H727/MEK cells show cross-resistance with the other ERK inhibitors (Fig. 1 panel b).

### Pharmacological characterization of resistant cells

**Fig. 2:**
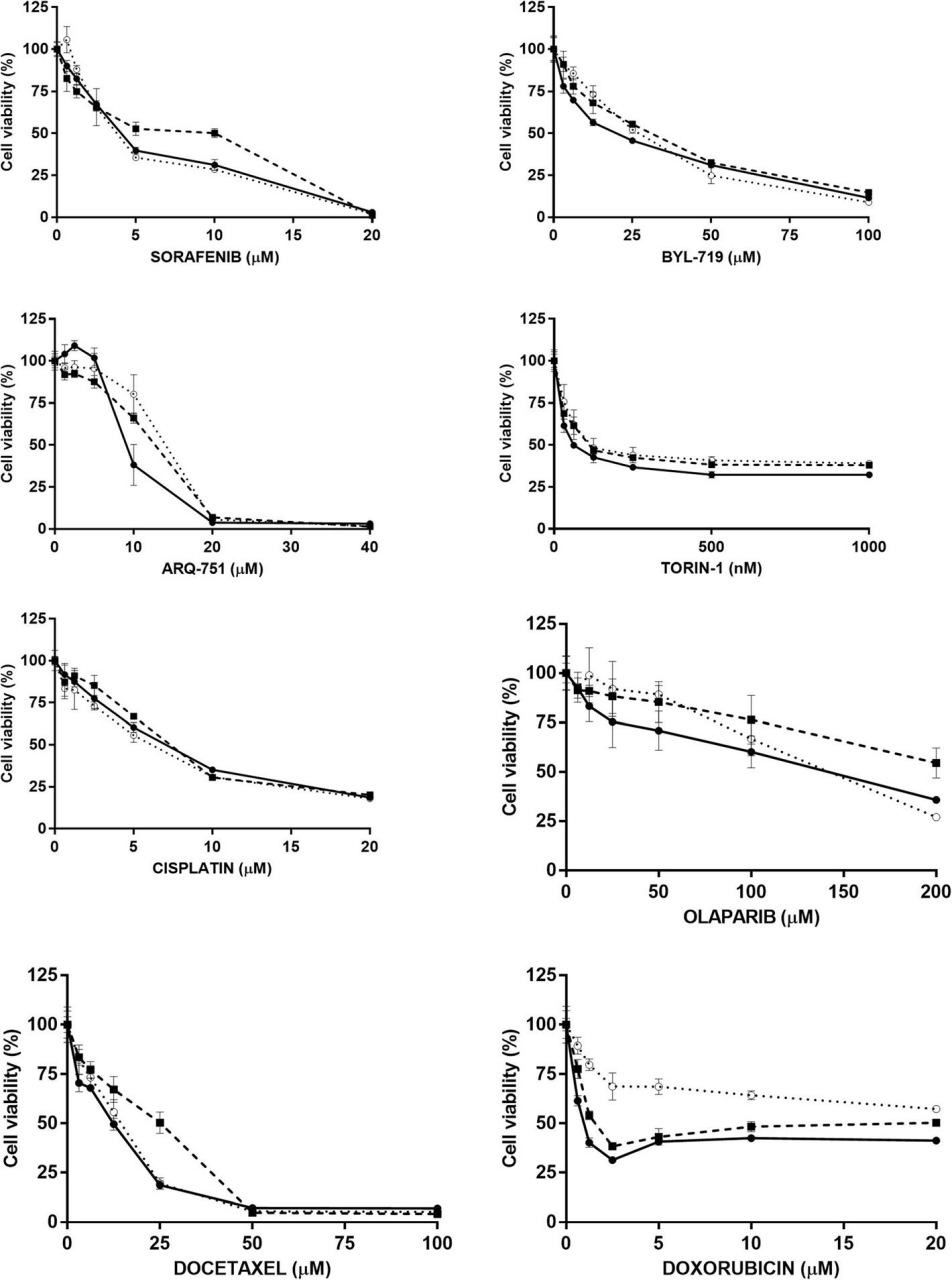
Activity of different anticancer agents against parental H727 (●—●), H727/MEK (■---■) and H727/SCH (○∙∙∙∙○) cells. Each graph report in the Y axis the % of controls at different concentrations as indicated for each drug in the X axis. Each value represents the mean of 6 independent replicates

The two cell lines were subsequently tested for their cross-resistance with other anticancer agents with different mechanism of action. As reported in Fig. 2, sorafenib (which inhibits B-RAF, upstream of MEK and ERK) had the same activity in parental H727 and in H727/MEK or H727/SCH resistant cells. The same holds true for three drugs acting on PI3K/akt/mTOR axis. BYL-719, specific PIK3CA alpha inhibitor, ARQ-751, an allosteric inhibitor of akt and torin-1, inhibitor of mTOR, all showed similar concentration-dependent curves and IC50 values (Table 2) in the two resistant cells and in the parental one. We also selected drugs acting on targets unrelated to MEK and ERK such as cisplatin (a DNA damaging agent) Olaparib (a PARP inhibitor), Docetaxel (acting on microtubules) and Doxorubicin (a DNA intercalating and damaging agent). Again, cisplatin and docetaxel maintained their activity in both sensitive and resistant cells. A slightly reduced activity for Olaparib was observed in both resistant cells, although the IC50 values were only slightly higher than that achieved in the parental cells. Finally, doxorubicin showed cross-resistance only in the H727/SCH subclones, while its activity in the H727/MEK subline was similar to that found in the parental H727.

**Table 2:** IC50 values of SCH772984 and MEK162 in parental and resistant cells

CELL LINE /DRUG	H727	H727-MEK	RI	H727-SCH	RI
SORAFENIB (μM)	4.07 ± 0.39	4.93 ± 0.69	1.2	3.97 ± 0.60	0.9
BYL 719 (μM)	16.98 ± 1.52	25.57 ± 1.72	1.5	24.36 ± 1.21	1.4
ARQ 751 (μM)	9.74 ± 0.62	11.50 ± 0.79	1.2	12.61 ± 0.48	1.3
TORIN-1 (nM)	73.13 ± 13.02	161.3 ± 29.49	2.2	203.7 ± 43.23	2.7
CISPLATIN (μM)	6.45 ± 0.25	7.01 ± 0.72	1.1	5.40 ± 0.49	0.8
OLAPARIB (μM)	126.1 ± 16.75	296.8 ± 65.53	2.3	131.8 ± 7.83	1.0
DOCETAXEL (μM)	9.72 ± 1.24	18.17 ± 2.99	1.8	12.02 ± 1.05	1.2
DOXORUBICIN (μM)	0.94 ± 0.48	2.1 ± 0.55	2.2	15.73 ± 8.72	16.7

Values are mean +/− SD; *RI* Resistance Index (Ratio of IC50 in resistant cells and in parental cells)

### Molecular characterization of resistant cells

**Fig. 3:**
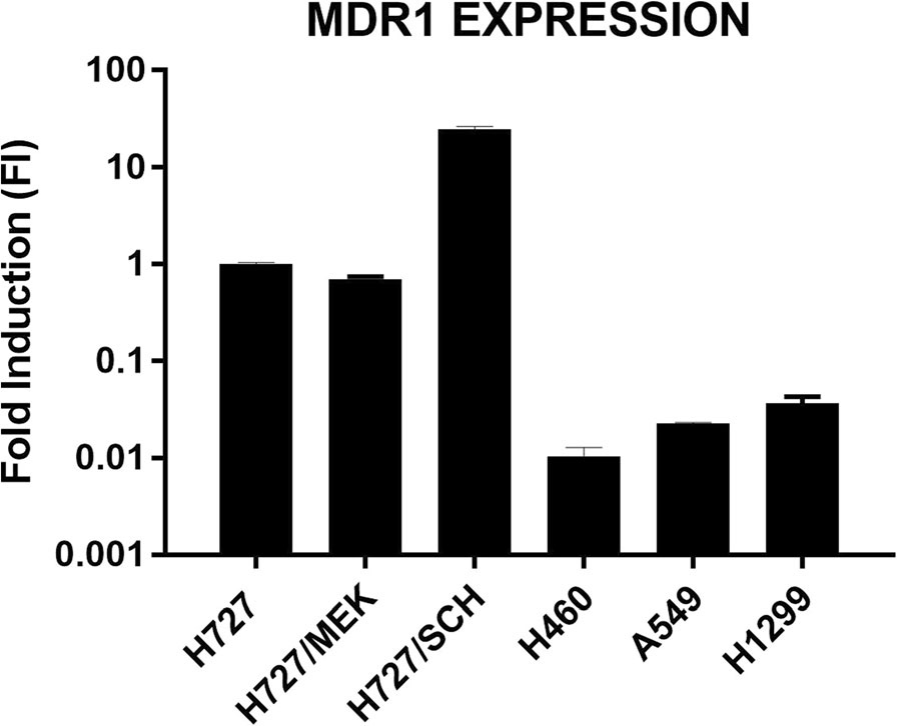
Expression of MDR-1 mRNA detected by RT-Real Time PCR in parental (H727) and resistant (H727/MEK and H727/SCH) cells. For comparison, the MDR-1 expression in three additional NSCLC cell lines (H460, A549 and H1299) is reported

The two resistant cell lines were tested for the expression of MDR-1. By RT-Real time PCR we detected the gene expression in parental and resistant clones and, for comparison in three additional NSCLC cell lines (NCI-H460, A549 and NCI-H1299). As reported in Fig. 3, H727/SCH showed an increased expression of MDR-1 mRNA relative to parental H727 cells while this was not true for H727/MEK cells. H727 parental cells already expressed 50–100 fold more MDR-1 mRNA than other NSCLC cell lines.

We then checked the downstream target modulation in parental and resistant cells after treatment with SCH772984. We treated all the three cell lines (H727, H727/SCH and H727/MEK) with the IC50 of SCH772984 in H727 cells (135 nM) and with a concentration 5 times higher (5x IC50, 635 nM). Extracts were taken after 6 and 24 h of treatment.

**Fig. 4:**
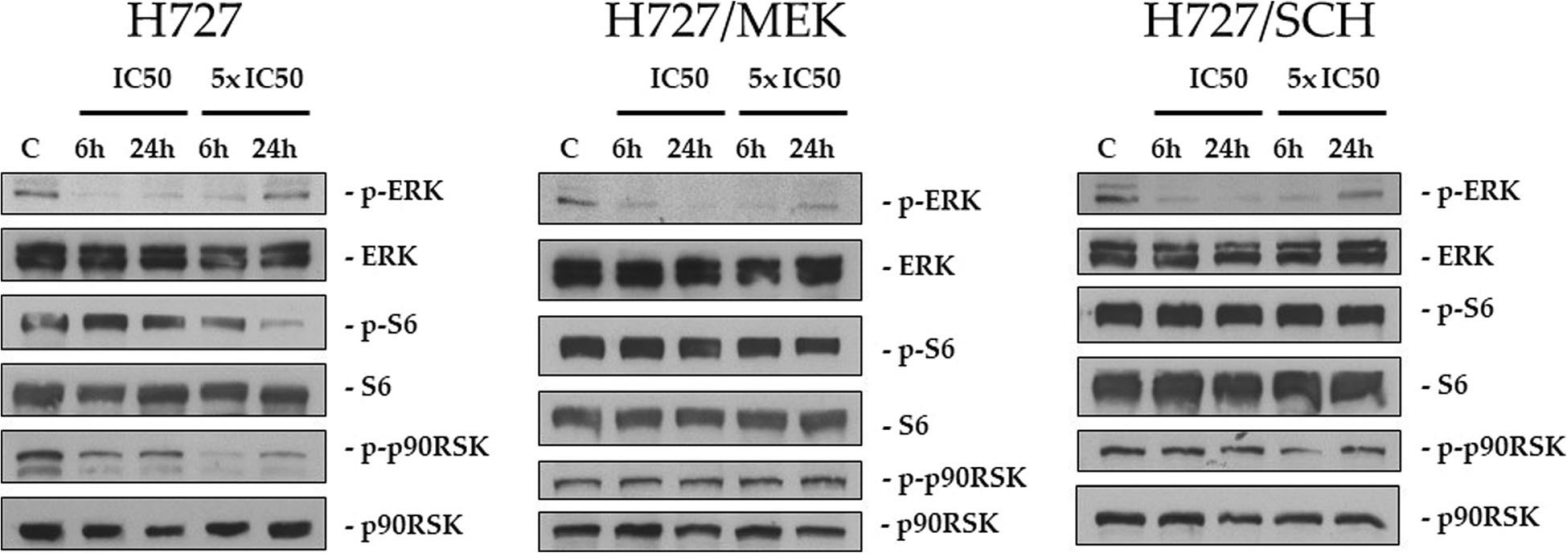
Representative western blot analysis showing the ability of SCH772984 to modify the phosphorylation of ERK and other proteins as indicated in the figure in parental H727 cells and in the two MEK and ERK resistant sublines 6 and 24 h after treatment. Cells were treated with a concentration corresponding to the IC50 of the drug in the parental cells and with a concentration 5 times higher (5xIC50)

SCH772984 was able to induce a similar downregulation of phosphorylated ERK in parental and in MEK or SCH resistant sublines (Fig. 4). In all the three cell lines, the maximal activity was observed at 6 h, while at 24 h the re-appearance of the phosphorylated form of ERK was appreciable, particularly with the highest drug concentration. In the parental cells, the drug was able to induce a decrease in phosphorylation of S6 and RSK, while this phosphorylation remained unaffected by treatment both in H727/MEK and in H727/SCH cells.

## Discussion

Almost invariably, drugs specifically targeting kinases (TKI) in clinical use developed resistance, which strongly affects their potential use. As examples, drugs targeting EGFR kinases (gefitinib, erlotinib or afatinib), both as reversible or irreversible ATP competitors, while producing very high response in patients with EGFR mutated NSCLC, with a significant increase in Progression Free Survival (PFS), the majority of patients relapses with a tumor no more responsive to these drugs [19–21]. The same is true for ALK inhibitors [22]. In both cases several mechanisms of resistance have been proposed, the main being the development of secondary mutations altering the binding of the drugs to the ATP-binding pockets of the kinase domain of the targets [23–25]. Fortunately, for these drugs, third and fourth generations compounds have now been synthetized and have shown activity in first generation TKIs-resistant tumors thus significantly increasing survival of patients with NSCLC [26, 27]. Similarly, for other tumors, B-RAF inhibitors (such as vemurafenib) showed impressive complete responses in melanoma patients which are challenged by the appearance of relapsing tumors resistant to these drugs [10, 16, 28] .

From these and other examples, it is therefore important to have models to study drug resistance for new drugs approaching the clinic. Here we studied in particular MEK and ERK inhibitors the first, in clinical use for the treatment of NSCLC [29, 30] and the second just entering initial clinical evaluation [5]. We were able to select, starting from a human NSCLC cell line sensitive to both ERK and MEK inhibitors, two cell lines with a stable resistance to the drugs. The cell lines showed cross-resistance one to each other but not to drugs targeting the same pathway upstream (such as sorafenib). It is not surprising that MEK and ERK inhibitors are cross-resistant each other, being their modulation of downstream targets (S6 and RSK) similar, however, we do not have, at present, an explanation for the lack of cross resistance with the B-RAF inhibitor sorafenib. In fact, it would have been expected cross resistance also for this drug. We can only speculate that inhibiting B-RAF could lead to (in addition to the canonical downstream proteins) alteration in other pathways not altered in H727/MEK and H727/SCH cells, thus justifying its activity. We can not exclude that sorafenib could act in these cells with a mechanism not related to B-RAF inhibition.

The two cell lines generated here did not show cross-resistance with drugs acting on parallel pathways such as the PI3K/akt/mTOR being the three inhibitors tested for the three main players of the pathway (i.e. PI3K, akt, mTOR) similarly active against sensitive and resistant cells. The resistant cells seem also not to have defects in DNA repair, at least as judged from the results obtained with the DNA damaging agents or DNA repair inhibitors tested here. A special note should be done for doxorubicin. This drug, in fact, showed a certain degree of cross-resistance in the H727/SCH cell line but not in the H727/MEK cell lines. We attribute this to the evidence that H727/SCH cells have a higher expression of MDR-1 gene compared to both parental H727 and H727/MEK cells. Being doxorubicin a drug substrate for MDR-1 the results are not surprising. We do not have an explanation for the higher MDR-1 expression observed in the H727/SCH cells. What we can reasonably exclude is that SCH772984 is a substrate of MDR-1. In fact, the parental H727 cells already express high levels of MDR-1, significantly higher than other NSCLC cell lines. However, H727 cells are very responsive in vitro to SCH772984 while the other NSCLC cell lines are not (both for A549 and H460 cells the IC50 of SCH772984 is > 10.000 nM, compared to the value of 135 nM in H727 cells). In addition, the similar downregulation of ERK phosphorylation shown in the present manuscript at equimolar concentration of SCH772984 in sensitive and resistant cells, indicates that the drug efficiently reaches the target inside the cells.

At molecular levels, we showed evidence that in resistant cells (both to MEK and ERK inhibitors) drug treatment did not induce a decrease in S6 phosphorylation, an event mediated by the RSK kinase, while this was clearly observable in parental cells. The involvement of S6 in resistance was recently shown in melanoma cells resistant to MEK inhibitors [12]. In these cells, RSK phosphorylation was unaffected while here, treatment of both H727/SCH and H727/MEK did not result in decreased phosphorylation of RSK (which was evident in parental H727 cells) suggesting that the cellular context can induce different molecular alterations.

## Conclusions

In conclusion, we have generated two NSCLC sublines resistant to MEK and ERK inhibitors, which show no cross-resistance to BRAF inhibitors and no cross-resistance to the agents inhibiting the parallel pathway PI3K/akt/mTOR. A lack of downregulation of RSK mediated S6 phosphorylation was observed in both resistant cells after treatment suggesting that this, as already shown in melanoma cells could be an important determinant of sensitivity to MEK and ERK inhibitors.

These cells, which show class-specific resistance, will be important for the development of new agents and combination able to circumvent resistance to MEK and ERK inhibitors. Although we did not test yet if the in resistance obtained in vitro can be confirmed in vivo too, we have several examples showing that MDR-1 unrelated drug resistance (as in this case) is maintained when cells are implanted in vivo [18, 31, 32]. Being ERK inhibitors at their initial clinical testing [5], the availability of models to study resistance in vitro and in vivo is of particular relevance.

## Abbreviations

DMSO: Dimethylsulfoxide
IC50: Concentrations inhibiting the growth by 50%
MDR-1: Multidrug resistance gene-1
NSCLC: Non-small-cell-lung-cancer
PFS: Progression Free Survival
STR: Short tandem repeat
TKI: Tyrosine kinase inhibitor
